# Key elements of cellular senescence involve transcriptional repression of mitotic and DNA repair genes through the p53-p16/RB-E2F-DREAM complex

**DOI:** 10.18632/aging.204743

**Published:** 2023-05-22

**Authors:** Renuka Kandhaya-Pillai, Francesc Miro-Mur, Jaume Alijotas-Reig, Tamar Tchkonia, Simo Schwartz, James L. Kirkland, Junko Oshima

**Affiliations:** 1 Department of Laboratory Medicine and Pathology, University of Washington, Seattle, WA 98195, USA; 2 Systemic Autoimmune Diseases Research Unit, Vall d’Hebron Research Institute (VHIR), Barcelona 08035, Spain; 3 Drug Delivery and Targeting Group, Clinical Biochemistry Department, Vall d’Hebron Hospital, Barcelona 08035, Spain; 4 Robert and Arlene Kogod Center on Aging, Mayo Clinic, Rochester, MN 55905, USA; 5 Department of Physiology, Mayo Clinic, Rochester, MN 55905, USA; 6 Department of Medicine, Mayo Clinic, Rochester, MN 55905, USA

**Keywords:** cellular senescence, cell cycle, DREAM complex, DNA repair

## Abstract

Cellular senescence is a dynamic stress response process that contributes to aging. From initiation to maintenance, senescent cells continuously undergo complex molecular changes and develop an altered transcriptome. Understanding how the molecular architecture of these cells evolve to sustain their non-proliferative state will open new therapeutic avenues to alleviate or delay the consequences of aging. Seeking to understand these molecular changes, we studied the transcriptomic profiles of endothelial replication-induced senescence and senescence induced by the inflammatory cytokine, TNF-α. We previously reported gene expressional pattern, pathways, and the mechanisms associated with upregulated genes during TNF-α induced senescence. Here, we extend our work and find downregulated gene signatures of both replicative and TNF-α senescence were highly overlapped, involving the decreased expression of several genes associated with cell cycle regulation, DNA replication, recombination, repair, chromatin structure, cellular assembly, and organization. We identified multiple targets of p53/p16-RB-E2F-DREAM that are essential for proliferation, mitotic progression, resolving DNA damage, maintaining chromatin integrity, and DNA synthesis that were repressed in senescent cells. We show that repression of multiple target genes in the p53/p16-RB-E2F-DREAM pathway collectively contributes to the stability of the senescent arrest. Our findings show that the regulatory connection between DREAM and cellular senescence may play a potential role in the aging process.

## INTRODUCTION

In the early 1960s, Hayflick and Moorhead demonstrated that primary human cells exhaust their proliferative capacity after a limited number of cell divisions and enter a permanent state of cell cycle arrest termed replicative senescence [1]. When cells undergo stress, the development and characteristics of the senescent cell fate depend on factors such as the stimulus causing cellular stress, the cell type, severity of the insult, and time since damage. Such cells can 1) undergo transient cell cycle arrest or enter quiescence; allowing them to repair damage and re-enter the cell cycle; 2) permanently lose proliferative capacity and exit the cell cycle by going into senescence if their damage is severe or irreparable; or 3) be removed by apoptosis/necroptosis [2–4]. Senescence is a key cell fate process that curtails the proliferation of damaged or aging cells and imposes a permanent exit from cell cycle progression. A multitude of cellular stress signals can induce and accelerate senescence, including inflammatory cytokines, replicative stress, telomere shortening, DNA damaging agents, oncogenic and oxidative stress, and metabolic or mechanical stress [4–8]. Senescent cells display distinctly altered architecture, such as enlarged morphology, altered chromatin, and the development of a senescence-associated secretory phenotype (SASP), all of which contribute to maintaining the senescent state [9–11]. Chronic inflammation and persistent senescence can accelerate aging, promote fibrosis, cause endothelial dysfunction, and contribute to the development of many aging conditions [12, 13]. We previously established the cytokine-induced model of senescence via chronic exposure to TNF-α and reported transcriptional changes and the molecular mechanism associated with upregulated genes in TNF-α induced senescence [7].

The molecular machinery of senescence is highly coordinated and related to several upstream effectors and downstream consequences. Over the past decade, extensive research has been conducted into multiple features of senescent cells, yet the gene regulatory networks behind this complex cell fate are still not fully understood. Multiple signaling pathways including p38, JAK/STAT, mTOR, and NF-κB have been reported to control and regulate senescence [10, 11]. Irrespective of triggering stimuli, various stress-mediated senescence pathways appear to converge on two important tumor suppressor pathways: p53/p21 and p16/RB [14]. Successive cell divisions and DNA replication are fundamental mechanisms for DNA repair and maintaining cellular integrity. Cell cycle regulation is a precisely organized and tightly controlled process: cyclic expression of hundreds of genes within and between each phase of cell cycle progression is crucial for proper cell division and repair. Cells are equipped with a series of check points at various phases of the cell cycle to ensure precise progression [15]. DNA damage is a well-established hallmark of aging, which is directly linked to senescence and its associated inflammation [4]. Once cells recognize damage, activation of the p53/p21 pathway leads to the initiation of DNA repair mechanisms and expression of cell cycle regulators that detect and repair damage. E2F is an important downstream transcriptional factor in the p16/RB pathway that plays a vital role in cell cycle progression [16, 17]. Inhibition of cyclins leads to dephosphorylation of RB through p16, which in turn shuts down E2F transcription targets. E2F acts as both an activator and repressor of gene expression that controls many cellular events including proliferation, differentiation, apoptosis, and cell cycle arrest. The RB-E2F complex and DREAM complex are known to repress transcription of a multitude of cell cycle-dependent genes [15, 18, 19]. The initiation, execution, and maintenance of senescence is well orchestrated and tightly controlled and senescence, in turn, is linked to other fundamental aging processes [4, 20]. Identifying the gene networks underlying senescence might help in elucidating links to other aging processes and their clinical consequences and will pave the way to devising targeted therapies that may delay, prevent, alleviate, or treat multiple age-related illnesses. TNF-α is a key proinflammatory cytokine that plays an important role in senescence, aging, and inflammation [7, 9, 21, 22]. To understand the molecular connection between inflammation and senescence, we studied the chronic effects of TNF-α on endothelial cells and later compared this to replicative senescent cells. In this study, we conducted a detailed pathway analysis of genes downregulated during senescence in vascular endothelial cells. We compared replicative senescence to TNF-α-induced senescence and identified pathways that involve cell cycle checkpoint regulation, DNA repair, and chromatin assembly and organization, all of which are extensively altered in both types of senescence. Our study enables us to envisage a global picture of genes and pathways dysregulated during senescence and provides novel evidence for involvement of p53/p16/RB-E2F-DREAM repressional targets in this complex cellular process.

## RESULTS

### Shared downregulated profiles between replicative and TNF-α-induced senescence

**Figure 1:**
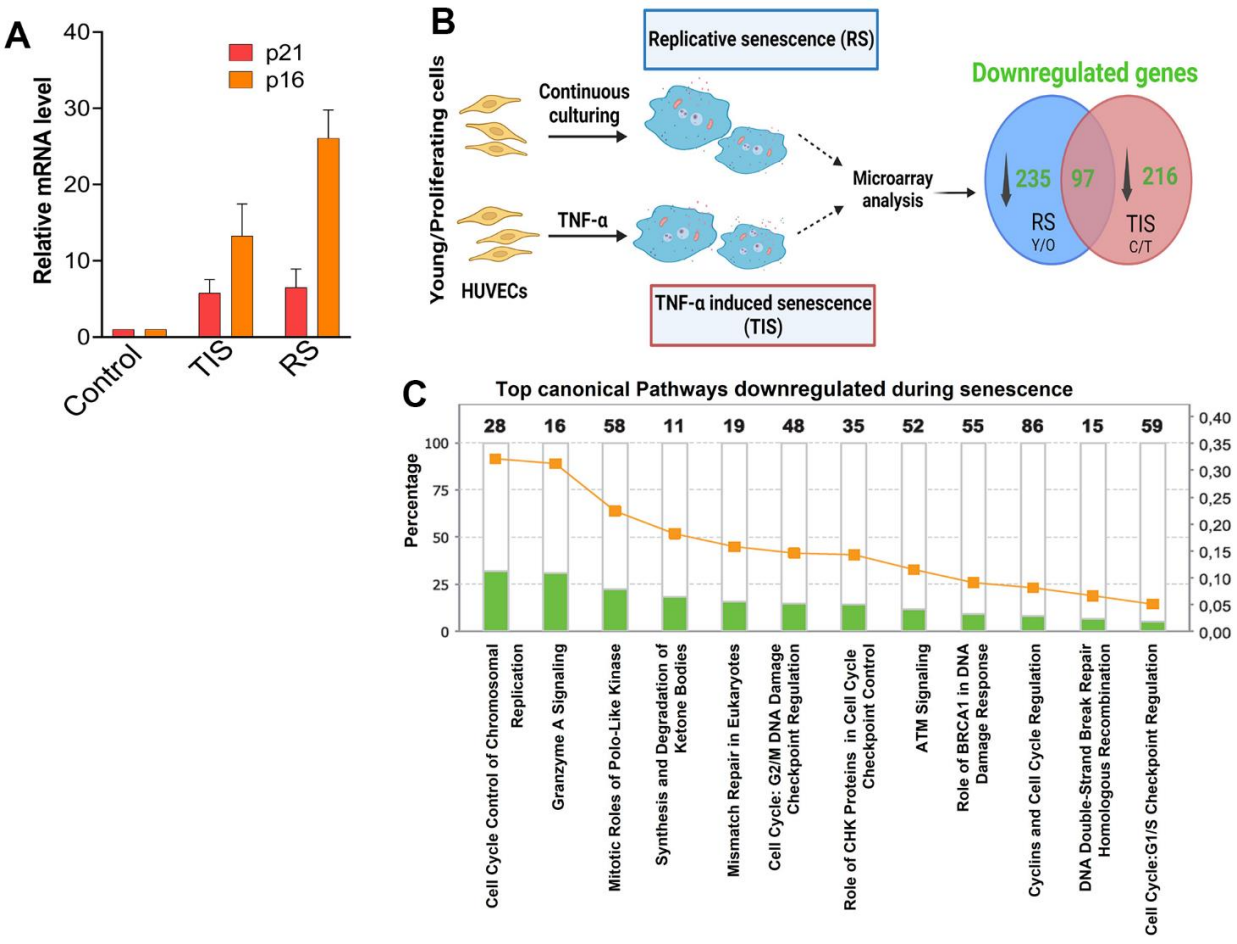
**Microarray analysis of replicative and TNF-α induced senescence.** (**A**) Expression of p21 and p16 in cells senesced by replicative stress or by exposure to TNF-α. (**B**) Experimental design for microarray analysis. Early passage or control HUVECs were continuously cultured until proliferative arrest to achieve replicative senescence (RS) or treated with the proinflammatory cytokine TNF-α (TIS) to achieve cytokine induced senescence. Gene expression changes of early passage control vs. replicative senescence and control vs. TNF-α-induced senescence were determined by microarray analysis. Venn diagram illustrating the number of genes downregulated in replicative senescence and TNF-α-induced senescence and overlap between the two groups (created with BioRender.com). (**C**) Top canonical pathways altered in senescence. Ingenuity Pathway Analysis (IPA) showing top canonical pathways associated to downregulated genes in senescence. The stacked bar chart displays the percentages of genes that were downregulated while the numerical value on top of each bar represents the number of genes associated with each canonical pathway. Green denotes downregulated gene expression.

Previously we studied pathways related to differentially upregulated genes in TNF-α induced senescence and reported the critical role of sustained expression of an inflammatory signature and JAK/STAT signaling [7]. To explore the pathways and networks that are repressed during senescence, we established replicative and TNF-α-induced models of senescence in HUVECs, as described in Figure 1 (Figure 1B). Senescence was confirmed by the expression of p21 and p16 by tracking BrdU incorporation, cell proliferation rate, and SA-β gal positive cells (Figure 1A and Supplementary Figure 1A–1C). To investigate pathways comprising downregulated genes in senescence, we compared replicative senescence to control (“early passage”) cells and TNF-α-induced senescence compared to controls (Figure 1B). We identified a total of 235 downregulated genes in replicative senescence and 216 genes in TNF-α-induced senescence, among which about 97 genes overlapped between both signatures (Figure 1B). The biological processes and canonical signaling pathways that these genes influence were explored using a functional core analysis based on IPA. We found that genes downregulated by both replicative senescence and TNF-α-mediated senescence shared similar functions and pathways. The most significantly altered pathways included cell cycle regulation, cellular assembly and organization, DNA replication, recombination and repair, cell growth and proliferation, and G/2M DNA damage checkpoint regulation (Figure 1C). This remarkable overlap of downregulated transcriptome signatures between replicative and TNF-α-induced senescence suggests that acceleration of senescence attenuates expression of similar gene targets.

### Senescent cells decline cell cycle and mitotic proteins

**Figure 2:**
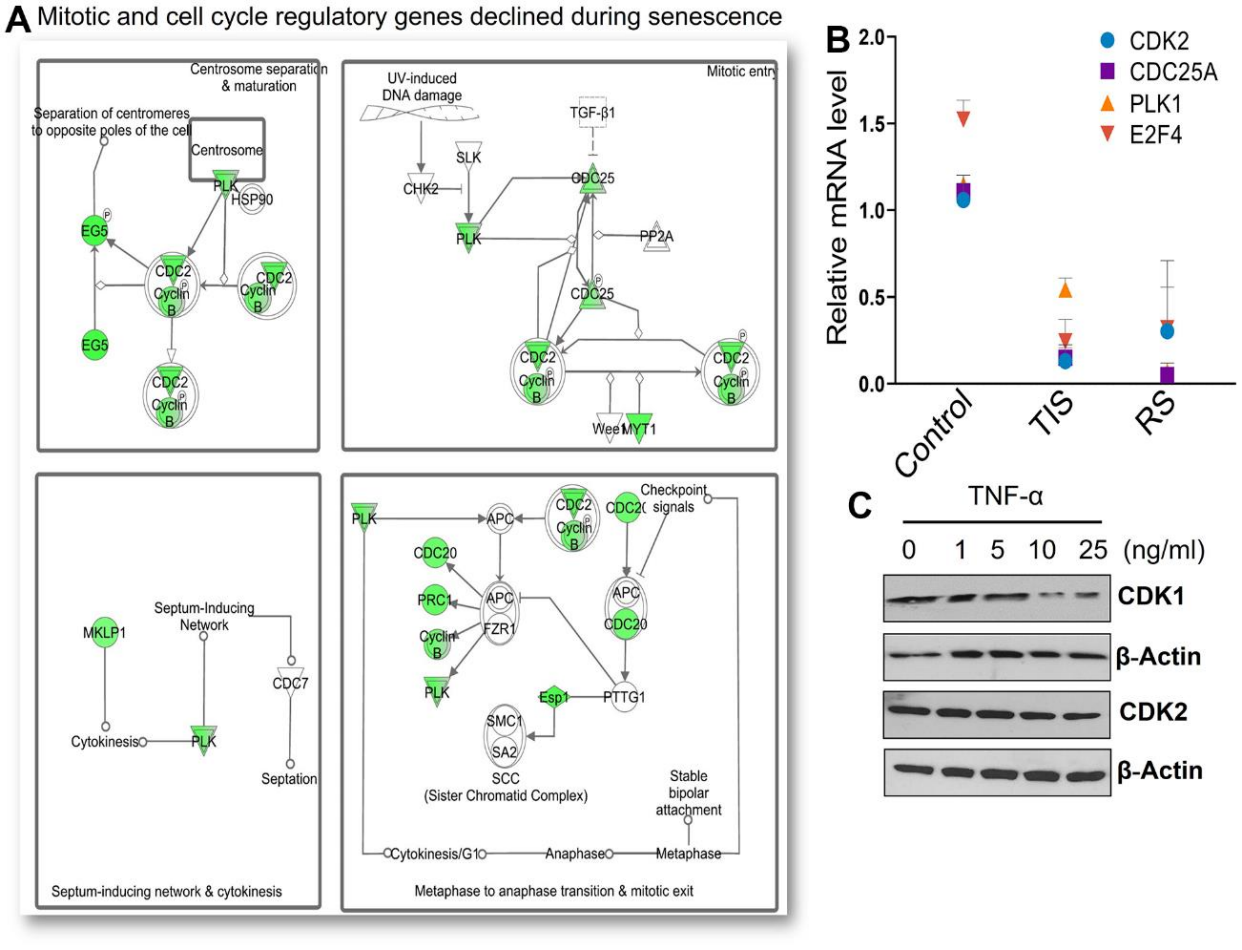
**Senescent cells display decreased mitotic gene expression.** (**A**) IPA analysis of significantly downregulated gene sets demonstrates many cell cycle dependent genes including cyclins and CDKs that are essential for G1/ and G2/M transitions. Many mitotic genes that play roles in check points, spindle assembly, and chromosome segregation were downregulated in senescence. (**B**) mRNA expression levels of selected targets including CDK2, CDC25, PLK1, and E2F4 were validated using RT-PCR. GAPDH levels were used for normalization. Means ± SD are presented in the graph. (**C**) Protein expression of selected cell cycle genes was validated by Western blot in cells exposed to different concentrations of TNF-α for 72 hours.

Cell cycle progression and regulation of mitosis require cooperative action of multiple cyclin-dependent kinases (CDKs) [23]. We identified that a considerable number of mitotic genes involved in cycle regulation, transitions, and progression were significantly downregulated in both replicative and TNF-α-mediated senescence. These included cyclins, CDKs that drive progression of G1/S (CCNA2, CCE2, CDC25A, CCB1, CCB2, CDK1, CDC25C, and FOXM1) and G2/M transition (CDC2, CDC20, and PLK1) (Figure 2A and Supplementary Figure 2 and Table 1). The cyclin E/Cdk2 complex and CDC25A are key G1/S phase regulators that inhibit phosphorylation of RB, blocking S phase entry, while expression of Cyclin A, Cyclin B, and CDK1 participate during the late S phase and G2/M [24, 25]. Reduced activity of multiple cyclins and CDKs, which play central roles in various phases of cell cycle progression, may partially explain the failure of DNA synthesis during senescence. The distinct roles of mitotic polo-like kinases (PLKs) during mitosis have been well established [26]. Here, we identified a significant decrease in a cluster of mitotic regulatory proteins that are important for mitotic entry, spindle assembly, and chromosomal segregation (Figure 2A). In addition, five genes (CDC25C, AURKB, PRC1, SMC4, and MAD2L1) that regulate mitosis and mitotic chromosome structure were also significantly decreased (Figure 2A). To validate the microarray data, the expression of selected genes was confirmed by RT-qPCR and Western blotting (Figure 2B, 2C). Other downregulated genes include CDKN3, ARHGAP11 variants A and B, SUV39H1, and CDAC variant genes, which are also involved in cell cycle regulation (Table 1). These data suggest that the lack of multiple cyclins and check point proteins that block cell cycle progression at different stages of the cell cycle contribute to cell cycle arrest.

**Table 1:** List of downregulated genes in replicative and TNF-α senescence.

**Function**	**Gene symbol**	**Replicative senescence**		**TNF-α senescence**	
		**p.value**	**Fold change**	**p.value**	**Fold change**
**Cell cycle regulation**	CCNA2	0.0039	-2.1	0.0092	-1.4
	CCNB1	0.0141	-1.7	0.0096	-1.6
	CCNE2	0.0085	-2.2	0.0031	-1.7
	CDC2	0.0032	-2.3	0.0009	-2.3
	CDC45L	0.0013	-2.5	0.0013	-1.8
	CDC25A	0.0140	-1.6	-	-
	CDC25B	0.0142	-3.2	0.0099	-1.5
	CDC25C	0.0012	-1.9	-	-
	CDKN3	0.0056	-2.3	0.0004	-2.7
	E2F8	0.0078	-2.2	0.0001	-2.5
	BUB1	0.0044	-2.2	0.0253	-1.5
**Mitotic/Checkpoints**	CENPE	0.0487	-1.7	0.4100	-1.2
	MAD2L1	0.0021	-2.4	0.0006	-2.5
	PBK	0.0047	-2.6	0.0083	-2.2
	PLK1	0.0018	-2.2	0.0100	-1.4
	PLK4	0.0208	-2.2	0.0068	-1.7
	CDC6	0.0018	-2.7	0.0014	-2
**DNA replication**	CDT1	0.0008	-2	0.0021	-1.3
	GINS1	0.0042	-2	0.0014	-1.9
	GINS2	0.0040	-2.1	0.0019	-1.9
	GINS3	0.0017	-2	-	-
	GINS4	0.0109	-1.6	0.0062	
	MCM2	0.0017	-1.96	-	-
	MCM3	0.0200	-1.5	0.0080	-1.92
	MCM4	0.0051	-1.74	-	-
	MCM5	0.0006	-2.3	0.0020	1.7
	MCM6	0.0094	-1.9	0.0009	-1.6
	MCM7	0.0009	-2.2	0.0008	-1.7
	MCM10	0.0007	-2.6	0.0051	-1.5
	MND1	0.0109	-1.8	0.0007	-2.2
	ORC1L	0.0005	-2.3	0.0120	-1.2
	RFC3	0.0043	-2	0.0031	-1.6
	RFC4	0.0046	-1.9	0.0352	-1.6
	TK1	0.0004	-2.7	0.0026	-1.7
**DNA repair**	EXO1	0.0028	-2.2	0.0222	-1.2
	FANCD2	0.0154	-2	0.0098	-1.6
	RAD51	0.0033	-2	0.0064	-1.6
	RAD51AP1	0.0014	-2.5	0.0037	-1.7
	TOP2A	0.0041	-2.2	0.0111	-1.5
	XRCC2	0.0147	-1.7	-	-
**Chromatin assembly, organization, and segregation**	HIST1H1A	0.0017	-2.5	0.0003	-2.6
	HIST1H1B	0.0023	-2.9	0.0023	-2.5
	HIST1H1E	0.0027	-2.6	0.0047	-2.1
	HIST1H1D	0.0152	-2.6	0.0150	-2.6
	HIST1H2AB	0.0332	-2	0.0264	-1.9
	HIST1H2AH	0.0204	-1.8	0.0262	-1.7
	HIST1H2AI	0.0067	-2.2	0.0085	-1.7
	HIST1H2AI	0.0078	-2	0.0053	-1.7
	HIST1H2BB	0.0211	-1.8	0.0256	-1.7
	HIST1H2AM	0.0081	-2.2	0.0289	-1.6
	HIST1H2BH	0.0084	-1.7	0.0028	-1.8
	HIST1H2BK	0.0197	-1.6	0.0169	-1.7
	HIST1H2BL	0.0033	-2.1	0.0028	-2.1
	HIST1H2BM	0.0019	-2.9	0.0080	-2
	HIST1H3A	0.0142	-1.8	0.0063	-2
	HIST1H3B	0.0066	-2.4	0.0038	2.2
	HIST1H3F	0.0034	-2.4	0.0104	-1.6
	HIST1H3H	0.0132	-1.7	0.0059	-1.5
	HIST1H3I	0.0363	-1.9	0.0028	-2.3
	HIST1H3J	0.0392	-1.8	0.0418	-1.6
	HIST1H4D	0.0261	-1.8	0.0128	-1.9
	HIST2H2AB	0.0107	-2	0.0039	-2.2
	HJURP	0.0033	-2.1	0.0026	-1.5
	HMGB2	0.0018	-2.2	0.0003	-2.1
	KIAA0101	0.0049	-2.1	0.0054	-2.5
	KIF15	0.0214	-2	0.0013	-1.7
	KIF18B	0.0005	-2.5	0.0026	-1.9
	KIF20A	0.0069	-2.2	0.0056	-2
	KIF22	0.0021	-1.8	0.0027	-1.5
	KIF2C	0.0047	-1.8	0.0072	-1.7
	KIF4A	0.0058	-2	0.0043	-1.5
	KIFC1	0.0022	-2.2	0.0031	-1.5
	MND1	0.0109	-1.8	0.0026	-2.4
	NCAPG2	0.0101	-1.9	0.0037	-1.9
	NDC80	0.0151	-1.9	0.0036	-2.2
	NUF2	0.0011	-2.8	0.0029	-1.9
	NUSAP1	0.0067	-2.3	0.0020	-2.1
	SMC4	0.0328	-1.8	0.0036	-1.8
	SPAG5	0.0016	-2.5	0.0002	-1.6
	SPC24	0.0045	-2.1	0.0002	-2.3
	SPC25	0.0021	-2.6	0.0002	-2.1

### Decreased expression of DNA replication, repair, and recombination genes

**Figure 3:**
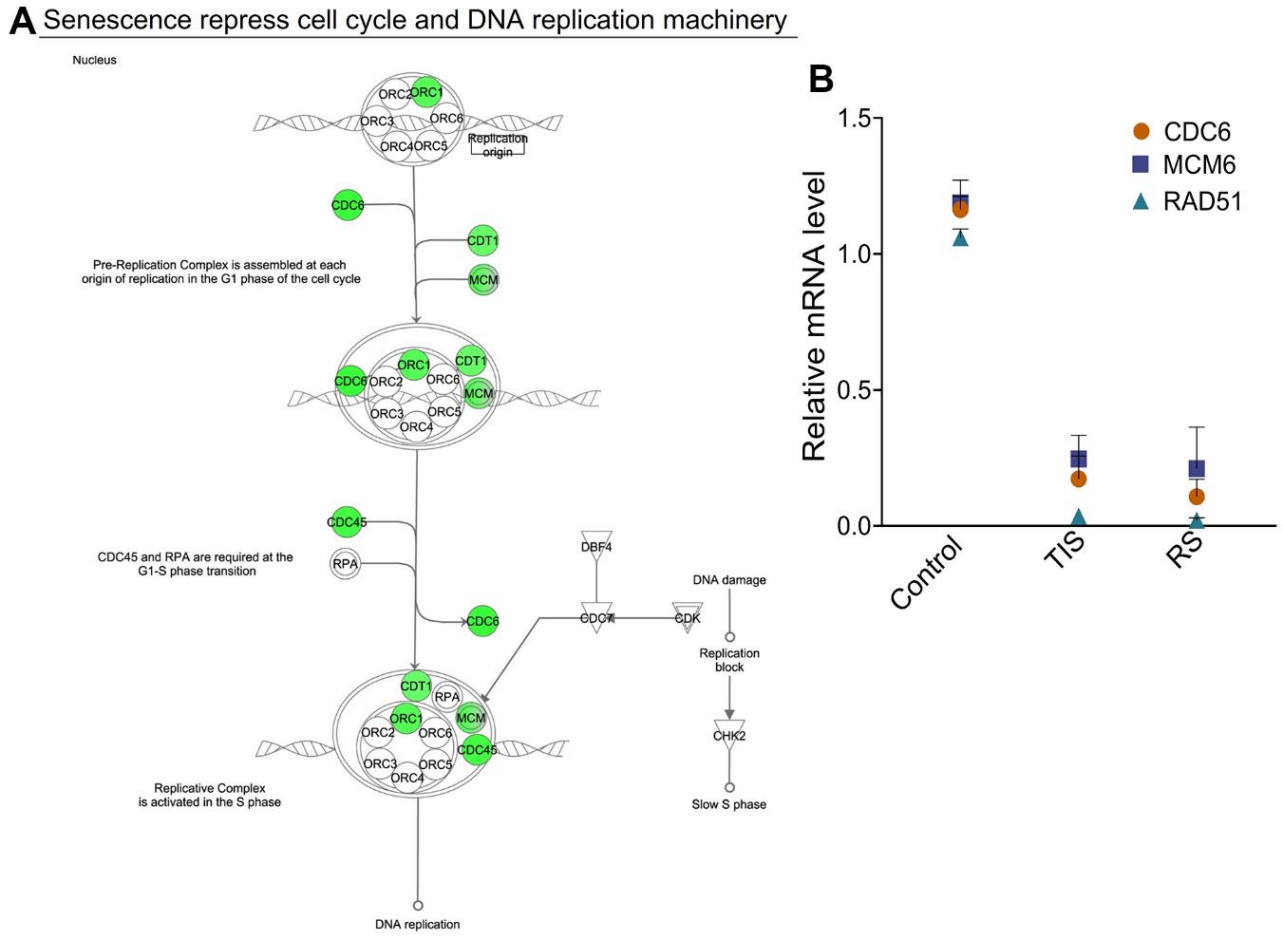
**DNA replication, recombination, and repair genes are repressed during senescence.** (**A**) Canonical pathway generated from the IPA analysis of the list of downregulated genes shows progressive changes of senescence-specific protein complexes associated with cell cycle control of chromosomal replication. Genes that are essential for pre-replication complex and initiation of DNA replication are decreased during senescence. Green denotes downregulated gene expression. (**B**) Expression levels of the genes RAD51, CDC6, and MCM6 were validated by RT-PCR in control, replicative senescence (RS) and TNF-α-induced senescence (TIS). GAPDH levels were used for normalization. Means ± SD are shown.

During aging, accumulation of DNA damage and deterioration of DNA repair mechanisms accelerate senescence [5, 27–29]. Based on IPA analysis, genes differentially downregulated in senescence were also associated with pathways that regulate DNA replication, repair, and recombination. The cell cycle control/chromosomal replication pathway is one of the most significant canonical pathways that was downregulated in both replicative and TNF-α-mediated senescence (ratio 9/28, P=4E-11 and 7E-11, respectively) (Figure 1C). Initiation of DNA replication requires the assembly of the pre-replication complex at the origin, and we found that many genes associated with the replication complex were decreased in senescence (ORC1, CDC6, CDC45, CDT1, C15orf42, MCM variants, and GINS1 complex variants) (Table 1). The canonical pathway generated from the IPA suggests that reduced expression of genes required for initiation and formation of pre-replication complexes in senescence blocks replication of DNA (Figure 3A). Efficient DNA repair and activation of DNA damage checkpoint proteins are essential for maintaining the integrity and stability of the genome [30]. The downregulated genes in the list of those related to senescence are associated with DNA damage checkpoints, DNA repair, and replicative stress, including EXO1, XRCC2, RAD51, AP1, RAD54L, TOP2A, BRAC2, FANCD2, and Replication Factor C variants 3 and 4. Reduced expression of replication and DNA repair genes suggests major roles in inducing senescence. The IPA further revealed that decreased expression of RAD51, FANCD2, EXO1, and RFC may contribute to stalled replication and defective DNA repair, especially homologous recombination, and mismatch repair (Supplementary Figure 3). The decreased expression of CDC6, MCM6, and RAD51 was validated by qRT-PCR (Figure 3B). Our results provide evidence for downregulation of multiple DNA repair and replication genes, thereby connecting senescence to impaired replication, compromised repair, and stalled progression of replication.

### Loss of chromatin structure and histone complex during senescence

**Figure 4:**
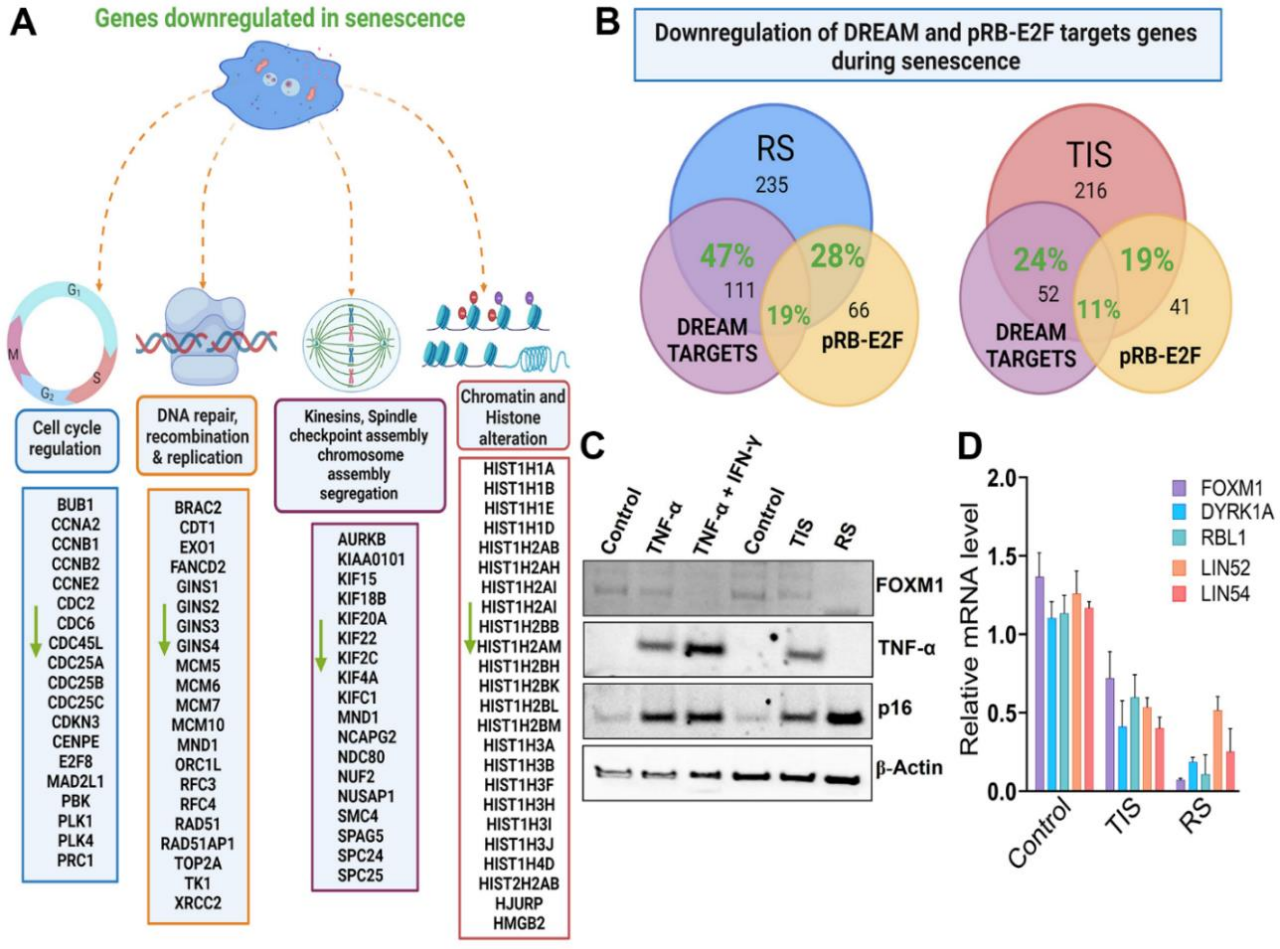
**Multiple targets of p53-DREAM/p16-RB-E2Fcomplex are stably repressed in senescent cells.** (**A**) Genes downregulated in senescence were grouped according to their cellular function, including cell cycle check point controls, DNA damage repair and replication, many histones, chromosome condensation, and kinetochore and spindle assembly proteins. (**B**) Overlap of downregulated genes in replicative senescence (RS) and TNF-α-induced senescence (TIS) that are linked to DREAM and RB-E2F targets. This Figure was created with BioRender.com. (**C**) Western blot analysis of FOXM1, TNF-α, p16 and actin in control, replicative senescence, TNF-α induced senescence or in cells exposed to combination of TNF-α/IFN-γ (**D**) mRNA expression levels of FOXM1, DYRK1A, RBL1, LIN52, and LIN54. GAPDH levels were used for normalization. Means ± SD are presented in the graph.

**Figure 5:**
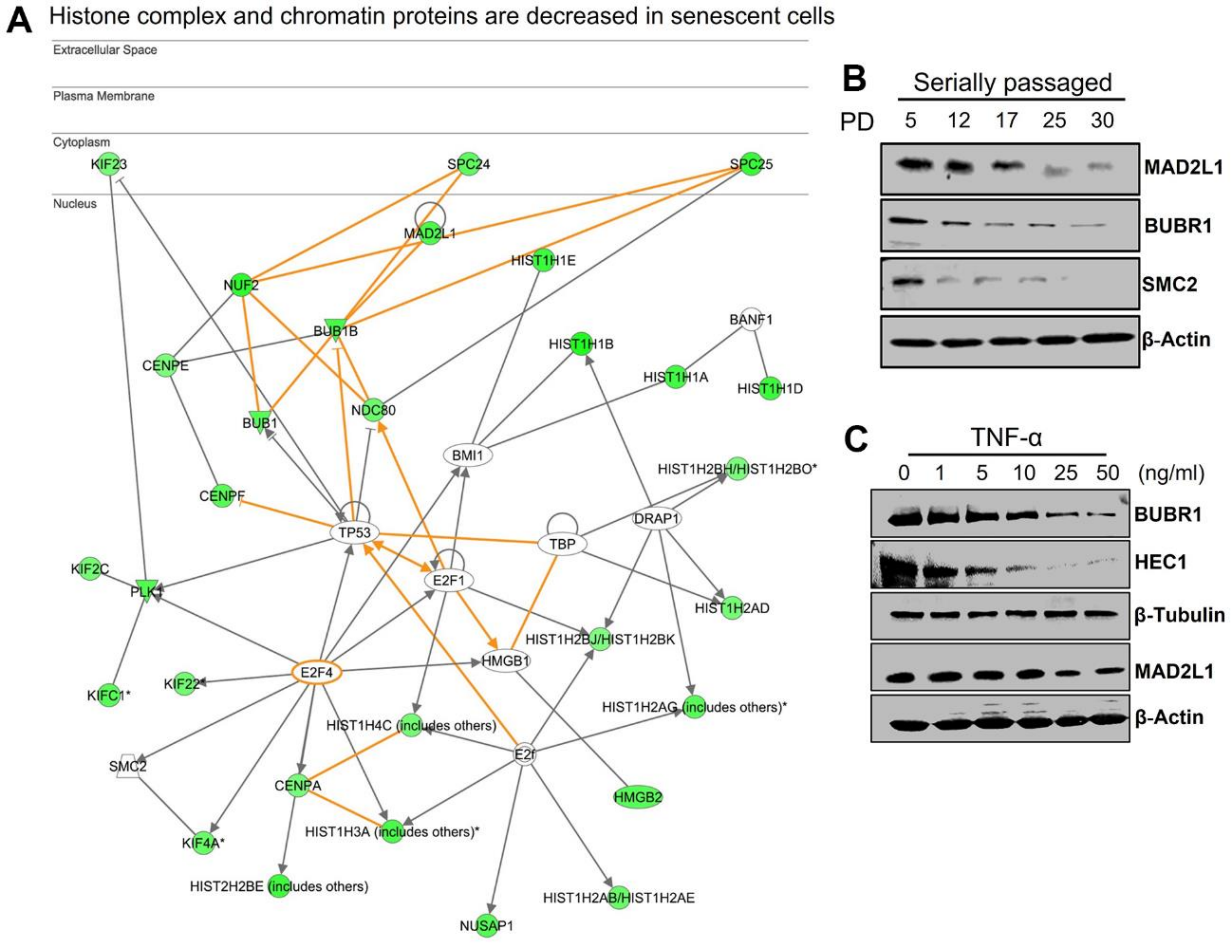
**Downregulation of networks of genes associated with chromatin structure.** (**A**) Network generated by IPA shows that multiple histones and genes associated with formation of chromatin structure were downregulated in senescence. (**B**, **C**) Western blot analysis of selected genes in serially passaged HUVECs cells (population doubling (PD) PD5-PD30) and TNF-α-mediated senescence (cell treated with various concentration of TNF-α). Actin and tubulin were used as loading controls.

Accumulation of DNA damage often increases mitotic errors, chromosome mis-segregation, and cellular senescence. Table 1 shows that a variety of genes that control chromosomal segregation, spindle assembly, and chromatin structure were perturbed in senescence. Among others, the mitotic markers PLK1, PRC1, and AURKB, which are known to play an important role in mitosis, chromosome condensation (SMC4), chromosome segregation (NDC80 or HEC1, CENPE, NUF2), mitotic check point regulation (MAD2L1, BUB1), and a cluster of kinesin family members, were downregulated (Figure 4 and Table 1). Profound heterochromatic changes and formation of senescence-associated heterochromatic foci (SAHF) are characteristic features of senescent cells [31]. We found altered expression of several genes involved in chromatin regulation, chromosomal segregation, and mitotic assembly. These include a cluster of genes that regulate segregation of chromosomes such as NDC80, BUB1, CENPE, HJURP, NUSAP1, ESLP1, SMC4, and CCA2, as well as six novel genes that regulate mitotic spindle formation, elongation, and assembly: KIF23, PRC1, SPC25, BIRC5, KIF2C, and NUF2 (Figure 5A). Global changes in heterochromatic structure influence senescence and normal aging processes [32, 33]. We also found that several linker H1 histones, which are essential for chromatin stabilization and mitotic chromosome architecture, were depleted in both replicative and TNF-α-mediated senescence. Western blot analysis indicated decreased expression of BUBR1, MAD2L1, SMC2, and HEC1 in replicative and TNF-α-induced senescence (Figure 5B, 5C). This data overall reflects global alterations of chromatin structure and genes that have direct roles in the shaping of this essentially irreversible state.

### Senescence involves transcriptional repression of p53-DREAM/p16-RB/E2F targets

A spectrum of genes that controls cytokinesis, chromosome assembly, spindle checkpoints, chromosome segregation, DNA synthesis and repair, and formation of chromatin structure was decreased in senescence (Figure 5A and Supplementary Table 1). Using network analysis, we found that many of these genes are connected to the p53/RB-E2F pathway (Supplementary Figure 4 and Figure 4A). p53 plays an important role in cellular stress responses and is known to transcriptionally repress many genes directly or indirectly through downstream activation of p21 [34, 35]. The dimerization partner, RB-like E2F and multi-vulval class B (DREAM), forms a p53-dependent transcriptional repressor complex that appears to control hundreds of cell cycle-dependent genes [18, 36, 37]. To further explore the targets of DREAM-RB/E2F, we utilized Target Gene Regulation Database 2.0 (accessed via http://www.targetgenereg.org [38] and overlapped our downregulated dataset with a publicly available list [18, 37, 39–42]. We identified that a large proportion of down modulated genes in both replicative and TNF-α-induced senescence were targets repressed by DREAM and RB/E2F (Figure 5B and Supplementary Table 1). We determined that 47% of DREAM and 28% of RB/E2F targets were repressed in replicative senescence, while 24% of DREAM and 19% of RB/E2F targets were repressed in TNF-α-induced senescence (Figure 5B). We also found significant overlap between p53-DREAM and RB-E2F target genes in senescence (Figure 5B and Supplementary Table 1). These include the cell cycle progression genes, CDK1, CDC20, CDC25, Cyclin A2, and Cyclin E2, as well as genes involved in the initiation of the ORCL1, CDC6, and MCM replication complexes. Additionally, genes that are involved in repair (TOP2A, FANCD2, and RAD51), several mitotic spindle check point proteins (MAD2L1, NDC80, PRC1, PLK1, and AURKB), centromere proteins, kinesins, and cell cycle check point genes (CDC6 and CDC45) were identified as targets of both DREAM and RB-E2F. The lack of expression of central components of the DREAM complex, including the RB pocket proteins RBL1, MYBL2 (G1/S and G2/M progression), and FOXM1, which drive expression late phase cell cycle genes, indicates the highly coordinated role of the DREAM pathway in senescence. Figure 4C shows the expression of FOXM1 were decreased upon senescence, and this was associated with increased levels of senescence marker p16, while increased TNF-α expression was observed in cytokine-mediated senescence. The RB-E2F complex, an important downstream target of the p16 pathway, plays a major role in cell cycle progression. We found a high percentage of genes that are targets of p53-DREAM are also targets of p16/RB-E2F, indicating the cooperative action of p53/p16-RB pathway in regulating senescence (Figure 4B and Table 1). Similarly, we found reduced RNA expression of dual specificity tyrosine phosphorylation-regulated kinase 1A (DYRK1A) and multiple genes that play an important role in the assembly of the DREAM complex including FOXM1, RBL1, LIN52, and LIN54 during senescence (Figure 4D). The induction of senescence is tightly regulated by both p53 and p16/RB. Our data suggest that the DREAM complex is an important downstream effector of the p53 and p16/RB pathway, which are involved in repressing numerous genes associated with cell cycle regulation, progression, proliferation, and DNA-repair.

## DISCUSSION

Senescent cells are a hallmark of aging, and having an increased proportion of these cells with age is considered to be a major risk factor for many diseases [43]. Inflammation, aging, and senescence are highly complex and dynamic processes that involve numerous transcriptional changes, epigenetic modifications, and alterations in cellular metabolism. A deeper understanding of the mechanisms underlying these processes and senescence specific changes that occur at the transcriptional level could form a basis for developing strategies to delay aging. Our previous findings identified pathways associated with upregulated genes during TNF-α senescence and demonstrated that sustained activation of the JAK/STAT pathway is a key mediator of the senescence associated-inflammatory and interferon signature [7]. This study suggests that the transcriptome signature of senescent cells goes beyond cell cycle arrest, with expression of multiple genes, from cell cycle to DNA repair to chromatin structure, being coordinately repressed to stably lock cells into this essentially non-proliferative state. This is in line with previous findings indicating that decreased expression of mitotic genes, defective DNA repair, and lack of chromatin stability are characteristic features of senescent cells that contribute to permanent cell cycle arrest [11, 27, 32, 33, 44, 45]. Our findings afford molecular insight into genes that control senescence and provide evidence for several downstream effectors of the p53/p21-p16 pathway that may be feasible to target to control senescence and age-related changes. We found that decreased expression of multiple genes that are primarily involved in cell cycle regulation and chromatin organization were common to both replicative and TNF-α-mediated senescence. This suggests that, irrespective of the initial trigger, regulation of senescence impacts the expression of genes that regulate cell cycle progression and repair machineries. Initiation of mitosis and sequential regulation of cell cycle progression requires coordinated activities of cyclins, cyclin-dependent kinases (CDKs), and cyclin kinase inhibitors (CDKIs) (McDonald, E.R 2000). Moreover, the CDC phosphatases (CDC25C and CDC20), Cyclin A/B, and CDK partners are involved in controlling entry into mitosis. Thus, the decreased expression of these genes reflects dysregulated cell cycle machinery and a blockade of the cell cycle during the establishment of senescence. Although the pathways regulated by differentially enriched genes seem to vary in both cases (data not shown), the down-regulated genes appear to engage the common regulatory pathways that contribute to establishing the senescent phenotype. Faithful segregation of chromosomes during cellular division is a fundamental process in cellular replication [46]. Increased mitotic errors and mis-regulation of mitotic genes have been positively correlated with aging [45]. Depletion of the number of mitotic proteins, all of which play diverse functional roles in different phases of mitosis, drives extended or prolonged mitotic arrest in both replicative and TNF-α-mediated senescence.

**Figure 6:**
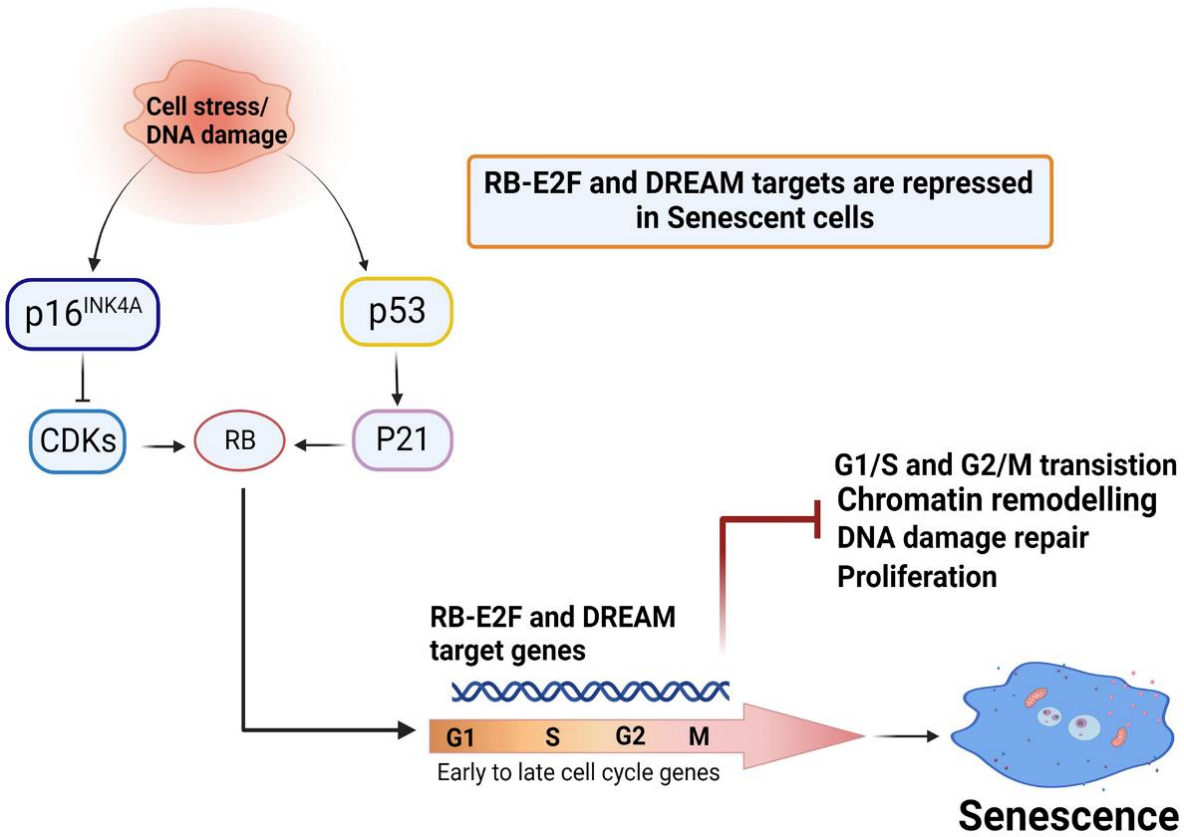
**Mechanisms proposed in this study.** p53/p21-p16/RB-E2F-DREAM repressor complex controls senescence. p16^INK4A^ and p53 are two master regulators of cellular senescence. Cell stress or DNA damage signals lead to activation p53 and transcriptional upregulation of the CDK inhibitor p21/CDKN1A, which in-turn hypophosphorylates RB. Activation of p16 ^INK4A^ controls phosphorylation of RB by binding to CDK4/6 and inhibiting the action of cyclin D. The active form of RB interacts with E2F to restrict transcription of genes that control cell cycle transition. RB-E2F and DREAM complex appear to cooperate to repress many genes that are required for G1/2 to G2/M transitions, potentially leading to impaired proliferation, defective DNA damage repair, altered chromatin, and establishment of senescence. (Created with BioRender.com).

Persistent DNA damage and defective repair processes have a role in the induction of senescence [5, 44, 47]. Subsequent disruption of DNA repair genes, such as Ku70, Ku80, MDC1, XRCC4, DNA ligase IV, BRAC1, and ERCCs, trigger senescence in both in cell culture and *in vivo* models [44, 48–50]. We observed decreased expression of several genes that are important for double stranded breaks, homologous recombination, and mismatch repair. This broadly supports the contention that execution of senescence acts as a failsafe mechanism to compromise defective repair machineries, thereby preventing catastrophic errors during replication. Nuclear DNA wrapped around histones is tightly packaged into chromatin, which profoundly influences DNA replication, transcription, repair, and recombination [51]. Furthermore, RB-E2F-mediated formation of heterochromatin and repression of DNA repair genes are considered to be hallmarks of senescence [33, 52]. Considering that SAHF formation, loss of H1 histones, and repression of heterochromatin genes are important for senescence induction [53], it is conceivable that sustained DNA damage with decreased DNA repair and altered chromatin structure contribute to the silencing of multiple RB-E2F targets to implement proliferative arrest. In other words, all these events may participate in repressing multiple genes that block various cellular functions, leading to extended mitotic arrest. A large body of studies has demonstrated the central role of p53 and p16/RB in senescence [6, 10, 11, 54]. Here, we provide evidence that replicative stress or cytokine-induced activation of p53/p21 and p16/RB co-operate with the DREAM complex to silence numerous genes required for progression of proliferation. Integrating meta-analysis findings and web-based atlases about p53-dependent regulation of human genes (http://www.targetgenereg.org), Kurt et al. reported that more than 250 targets of the p53-DREAM pathway are associated with cell cycle regulation. DREAM is considered as a master regulator of cell cycle-dependent genes [15], but the precise role of the DREAM complex in cellular senescence is still not well understood. Ruchi et al. reported the bypass of senescence with simultaneous expression of the DREAM components, MMB-FOXM1 [55]. Repression of FOXM1 during aging is known to trigger dysfunctional mitotic machinery and contribute to aneuploidy-driven senescence, while cyclic FOXM1 expression delayed senescence and alleviated aging phenotypes in progeroid mice [56, 57]. Disturbing DREAM complex assembly with small molecule kinase DYRK1A inhibitor reduced the ability of cells to undergo quiescence or senescence, while simultaneously improving proliferation in adult human pancreatic β cells [58, 59]. Furthermore, we found in contrast to TNF-α induced senescence, serum-starved HUVECs or quiescent 82-6 fibroblast cells 1) did not increase the expression of SASP genes such as IL-6 or IL-8 (Supplementary Figure 5A, 5B) or 2) senescence markers (data not shown). However, both senescent and quiescent cells decreased DREAM-associated genes DRYK1A and LIN52, these results indicate that the function of DREAM complex varies depending on the context. A recent report showed that the DREAM complex plays a role in the transcriptional repression of wide range of DNA repair genes in C. elegans [60]. Similarly, our data suggest that a broad range of genes repressed during senescence are connected to the DREAM complex and might be critical for cell cycle exit or mitotic decline. We suspect that during the initiation of senescence, transcriptional activation of p53 acts as an upstream regulator of many pathways, including p21. However, p53 may later function as a downstream effector that represses genes required for proliferation, acting both as an activator and repressor of execution of senescence. p53-mediated repression of its target genes occurs either by direct binding to consensus DNA sequences or, in the absence of DNA binding, repression of some p53 target genes requires activation of p21 [61]. Expression of p21 is known to prevent phosphorylation of RB, thereby resulting in the formation of RB-E2F complexes, which in turn actively represses E2F target genes [62]. Senescent cells can have increased expression of p16, which is known to control phosphorylation of RB by inhibiting cyclins and CDKs [63]. Presumably, the initial phases of senescence are regulated by p53, while feedback circuits mediated by the p16/RB-E2F pathway may be important for controlling DREAM assembly and the subsequent ability to stably maintain the arrested state. Interplay between p53 and p16/RB tumor suppressor pathways both in aging and senescence has been well documented. Uxa et al. demonstrated that in response to p53 activation, DREAM and RB cooperate to induce gene repression and cell cycle arrest [64]. A recent study found DREAM as a master regulator of somatic DNA repair genes, and pharmacological inhibition of DREAM de-repressed DNA repair genes and prevented DNA damage accumulation [60]. It is noteworthy that post-translational modifications of DNA repair factors are known to play a critical role in the DNA damage response as they can affect the activity, localization, and stability of DNA repair mechanisms. Further investigations are important to gain the complete picture of post-translational regulation of DNA repair genes during senescence. Our findings further extend and identify the participation of DREAM in controlling senescence and genes associated to chromatin structure and DNA repair. Future studies to investigate how p53-DREAM is coordinated with p16-E2F will provide more answers to this complex process. Our model suggests that, upon stress or DNA damage, activation of p53/p21, coupled with p16/RB, and DREAM leads to transcriptional repression of hundreds of mitotic genes to exit cell cycle, enter senescence, and remain arrested (Figure 6). Thus, highlighting the role of DREAM and its association with p53/p21-p16/RB pathway plays a key role in the maintenance of this complex senescent phenotype.

## MATERIALS AND METHODS

### Cell culture

Primary human umbilical vein endothelial cells (HUVECs) were purchased from Lonza (C2519). HUVECs were cultured in endothelial growth medium (basal medium-2; EBM-2; Lonza CC-3156) supplemented with Endothelial Cell Growth Medium (EGM)-2 Bullet Kit (CC-3162; Lonza). HUVEC monolayers were maintained in tissue culture flasks or culture plates at 37° C in a humidified incubator with 5% CO_2_ and 95% air [65].

### Experimental workflow

We have previously reported pathways associated with differential upregulated genes in TNF-α-induced senescence [7]. In this study, to characterize downregulated gene expression patterns of replicative and TNF-α-induced senescence, we compared the expression profiles of downregulated genes in replicative senescence to those in senescence induced by the inflammatory cytokine TNF-α. To determine the gene expressional changes, three independently cultured samples from early passage (PD 3-PD5) were compared to senescent passage (PD 28-35) (replicative senescence) and cells senesced by exposure to TNF-α (PD5-PD8) were compared to the corresponding control group (TNF-α induced senescence).

### Induction of cellular senescence

Senescence induction was performed as described previously for both the replicative and TNF-α-induced senescence [7, 22]. Replicative senescence was achieved by replication exhaustion. Briefly, 0.5x106 of HUVECs (Early passage 2 or p2) (PD3) seeded in T75 tissue culture flasks were cultured continuously until cells ceased proliferation and exhibited senescent morphology, or replicative senescence (Senescent passage p12-15) (PD 28-35). Cells were sub-cultured at 4–5-day intervals or after achieving 80% confluence. Cells were harvested, counted, and re-seeded with same number of starting cells in new culture flasks. Morphology of cells was routinely monitored, and photographs were taken. For TNF-α-induced senescence, HUVECs were chronically cultured in the presence or absence of 5ng/ml TNFα for 26 days, as described previously [7]. Cell numbers were ascertained at the end of each subculture. To determine gene expressional changes in replicative and TNF-α-induced senescence early passage or control HUVECs (PD 3-5) were compared with old passage or replicative senescent cells (PD 28-35) while control (untreated cells) (PD <5) was compared with TNF-α treated cells (PD 5-8).

### Gene expression profiling

Three biological replicates from each experimental condition were used for microarray analyses using the Affymetrix Human gene chip 1.0 ST array system, as described previously (Kandahaya-Pillai et al., 2017). The human gene chip gene 1.0st array interrogates 28,869 well-annotated genes with 764,885 distinct probe sets. Total RNA was extracted from the experimental samples using TRIzol® reagent (Invitrogen; Thermo Fisher Scientific, Inc.). RNA was isolated using the phenol-chloroform method, following which RNA purity was examined using a Bioanalyser 2100 (Agilent Technologies). RNA expression profiling was performed following the pico profiling method [66]. Array hybridization, washing, staining, and scanning of Affymetrix Human Gene ST 1.0 were performed according to the manufacturer’s recommendations. Briefly, cDNA library preparation and amplification were performed from 50ng of total RNA using WTA2 (Sigma-Aldrich) with 17 cycles of amplification. 10ng of cDNA were subsequently fragmented and biotinylated by terminal transferase obtained from the Genechip mapping 10kvs assay kit (Affymetrix). The labeled samples were hybridized to Human gene ST1.0 arrays and scanned with a Genechip scanner 3000. The array data were deposited in the GEO database (accession number GSE195517).

### RNA expression analysis

The CEL files were from DAT files using GCOS software. To generate log_2_ expression estimates, overall array intensity was normalized between arrays and the probe intensity of all the probes was summarized to a single value using the robust multichip average (RMA) algorithm [67]. Differentially expressed gene sets were then identified using classical parametric hypothesis testing of mean comparisons. To group differentially downregulated genes, we applied a stringent selection criterion with a threshold of fold > 1.5 and P-value >0.05 being considered significant. Gene filtering, pair-wise comparisons, t-tests, and PCA were conducted using Bio-conductor (http://www.bioconductor.org) [68] and the R package (http://www.rproject.org) [69].

### Network and pathway analysis

We used Ingenuity pathway analysis (IPA) software (Qiagen). Gene networks and canonical pathways representing key genes were identified using the IPA curated database as previously described [7]. Briefly, data sets containing differently regulated gene list with corresponding fold changes were uploaded into the web-based application. Each gene identifier was connected and mapped to its corresponding gene object in the ingenuity pathways knowledge base (IPKB). The gene data set was mined for significant pathways using the IPA library to map pathways, and networks were generated by using graphical representation of the molecular relationship between genes and gene products.

### Senescence-associated β-galactosidase assay (SA-β-gal)

SA-β-gal activity was detected using a senescence staining kit according to the manufacturer’s recommendations (Sigma-Aldrich), as described previously [65]. For SA-β-gal staining, cells were fixed in 2% formaldehyde/0.2% glutaraldehyde for 5 minutes, washed three times with phosphate-buffered saline (PBS), stained with SA-β-gal staining solution (at pH 6), and incubated overnight at 37° C without CO_2_. Stained cells were then visualized and photographed with a Nikon microscope. The percentage of cells positive for SA-β-gal was determined in duplicate in 200 cells per sample.

### BrdU incorporation

BrdU incorporation was measured using a BrdU Flow Kit (BD Biosciences) following the manufacturer’s instructions. As previously described, cells were labelled with 10μM BrdU for six hours. Cells were then harvested, washed once with 1x phosphate-buffered saline, and fixed in 100μl of Cytofix/Cytoperm for 30 minutes at room temperature. After DNase treatment, BrdU-incorporated cells were then stained with FITC-conjugated anti-BrdU antibody and 7-aminoactinomycin D. BrdU positive cells were analyzed using a FACS caliber flow cyto-meter (BD Biosciences). Data analysis was performed using FCS express software.

### Western blots

Protein extracts were prepared by eluting whole cell-lysates using 2x Laemeli sample buffer followed by sonication of samples for 10 seconds and stored at -20° C until use. All samples were boiled at 95° C for 5 min before loading. Samples were resolved by polyacrylamide gel electrophoresis and proteins were transferred onto nitrocellulose membranes. Membranes were then blocked for 1 hour in 5% milk/tris-buffered saline–1% Tween (TBST). Indicated primary antibodies were diluted in 5% milk/TBST or 5% BSA/TBST and incubated at 4° C with gentle agitation overnight. Blots were washed for a minimum of three times in TBST for more than 15 minutes. Secondary antibodies were prepared in 5% milk/TBST, and membranes were incubated at room temperature for 45 minutes. Protein bands were detected by Western blot using an ECL Detection Kit. Details of antibodies used in this study are listed in the Supplementary Figures (Supplementary Table 2).

### Quantitative reverse transcription polymerase chain reaction (qRT-PCR)

Total RNA was extracted from experimental samples using the phenol-chloroform method. For qPCR analysis cDNA was generated from 1.0 μg of RNA using a high-capacity reverse transcriptase kit (4368814, Thermo Fisher Scientific) according to the manufacturer’s instructions. mRNA gene expression was performed using Taqman Fast Advanced master mix and Taqman probes in an Applied Biosystem 7500. Samples were run in triplicate in all the experiments. The ΔΔCT method was used to quantify expression levels and GAPDH was used as the housekeeping gene to obtain relative mRNA expression. All the primers were obtained from Thermo Fisher Thermo Fisher Scientific and are listed in the Supplementary Table 3.

### Statistical analysis

Statistical assessments were performed using Prism 9.0 (Graph Pad). Three independent biological experiments were performed for all qPCR assays. Data represent the mean ±SD (standard deviation).

### Data availability

The datasets used and analyzed during the current study are available from the corresponding author on reasonable request. The microarray data of this study are deposited at GEO database and are available under accession number GSE195517.

## Supplementary Material

Supplementary Figures

Supplementary Table 1

Supplementary Tables 2 and 3
