# Supplementary material for: Key elements of cellular senescence involve transcriptional repression of mitotic and DNA repair genes through the p53-p16/RB-E2F-DREAM complex
Source: Aging (Albany NY). 2023 May 22;15(10):4012–34. doi: 10.18632/aging.204743 (PMC10258023; doi:10.18632/aging.204743)
Supplement: Supplementary Figures [file aging-15-204743-s001.pdf]

SUPPLEMENTARY FIGURES

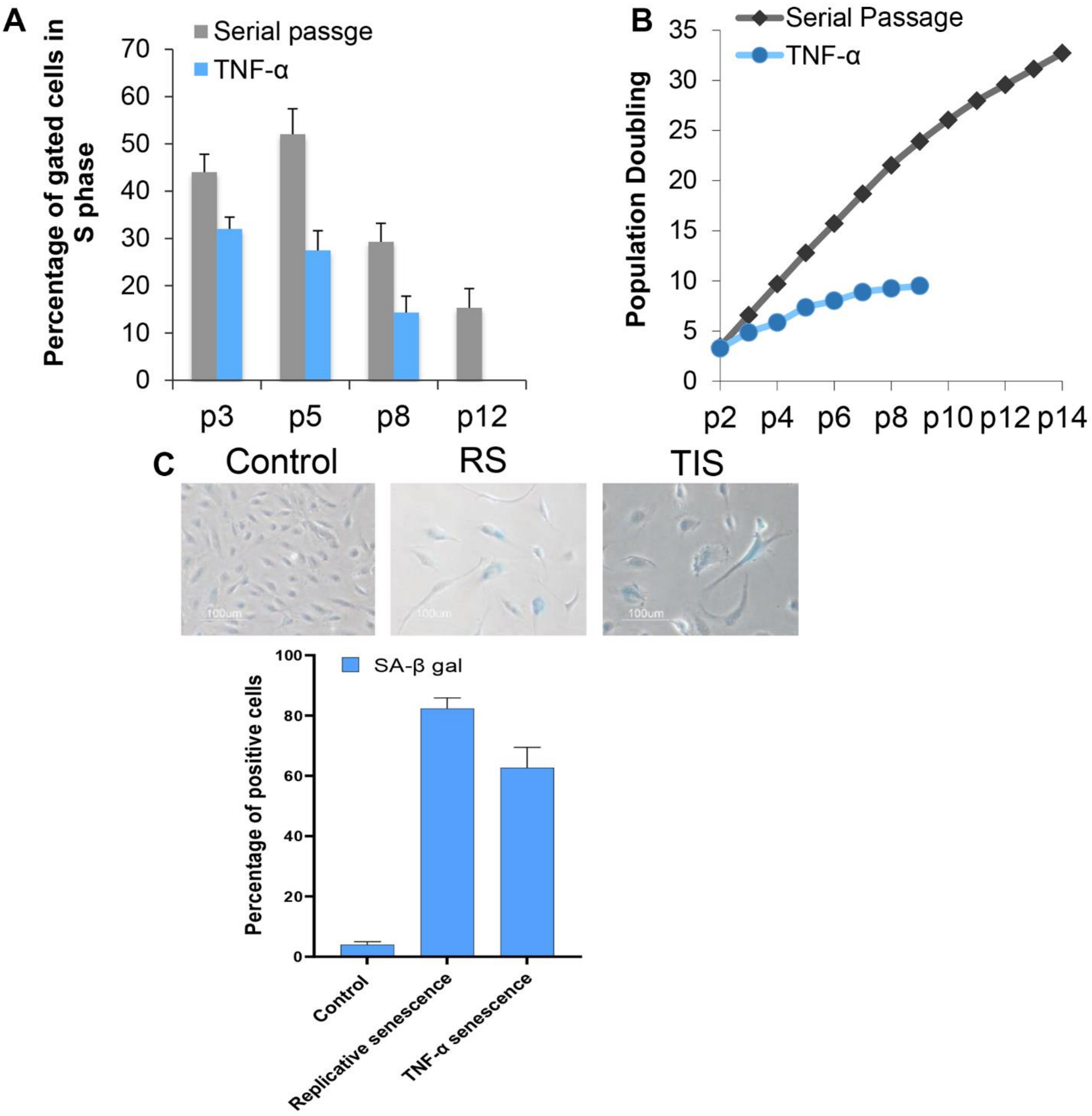

Supplementary Figure 1. Characterization of replicative and TNF-α senescence. (**A**) Percentage of S phase cells during senescence. BrdU labelled cells were stained and analysed by flow cytometry. Percentage of cells gated in S phase of the cell cycle were determined in serially passaged or in cells exposed to TNF-α. (**B**) Growth curve of HUVECs either serially passaged until replicative exhaust or proliferative arrest, or chronically treated with inflammatory cytokine TNF-α as indicated. (**C**) Representative images and percentage of senescence associated beta gal positive cells (SA-β gal) in control of early passage 3 (PD5) Replicative senescence passage p14 (PD34) or in cells chronically exposed to TNF-α (5ng/ml) passage 7 (PD 8).

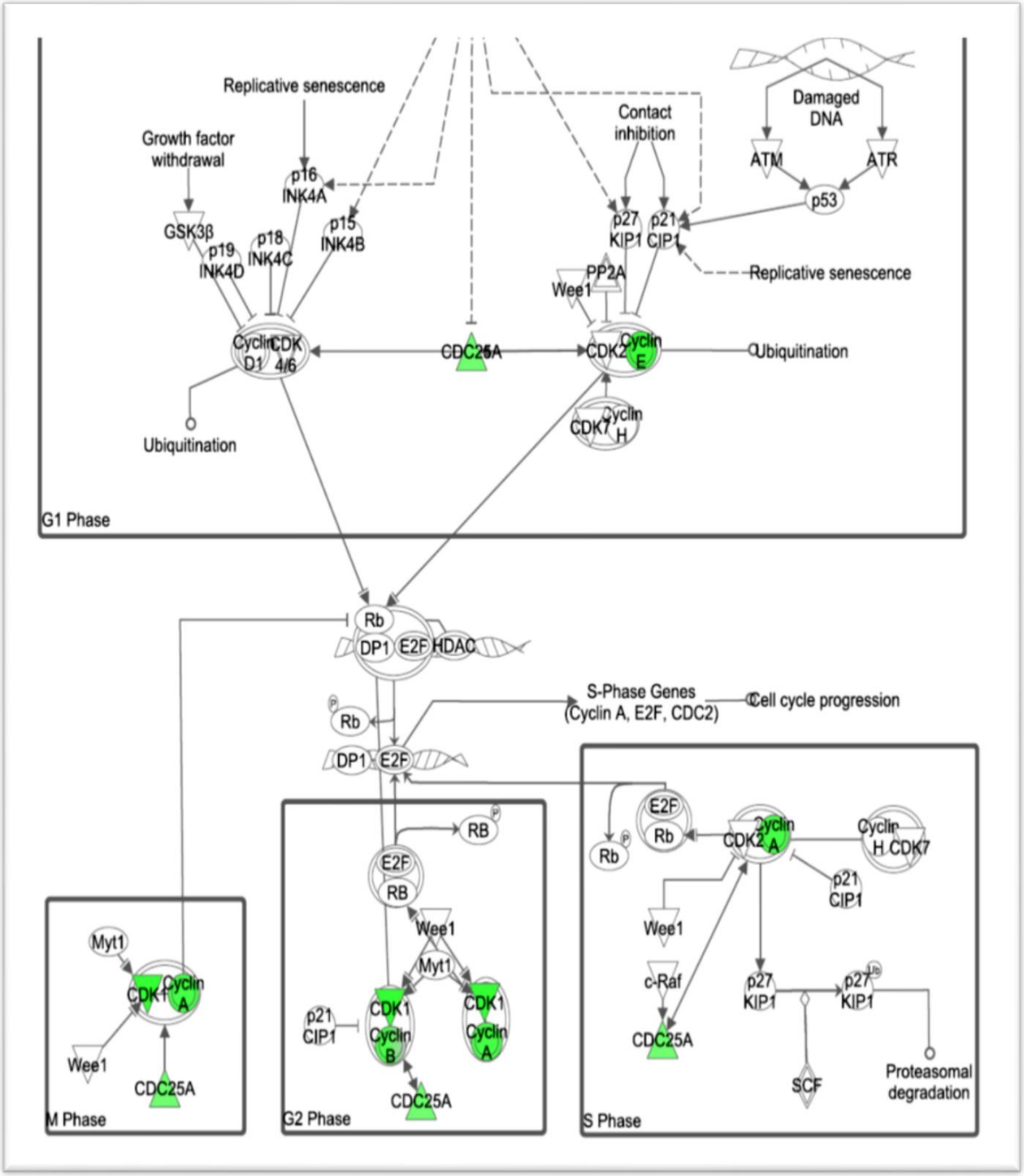

Supplementary Figure 2. Downregulation of cell cycle regulatory genes during senescence. Canonical pathway generated by IPA illustrates that the expression of Cyclins and CDKs which are essential for transition and regulation of G1/S and G2/M phase of the cell cycle were downregulated during senescence. Green color represents decreased expression of the genes.

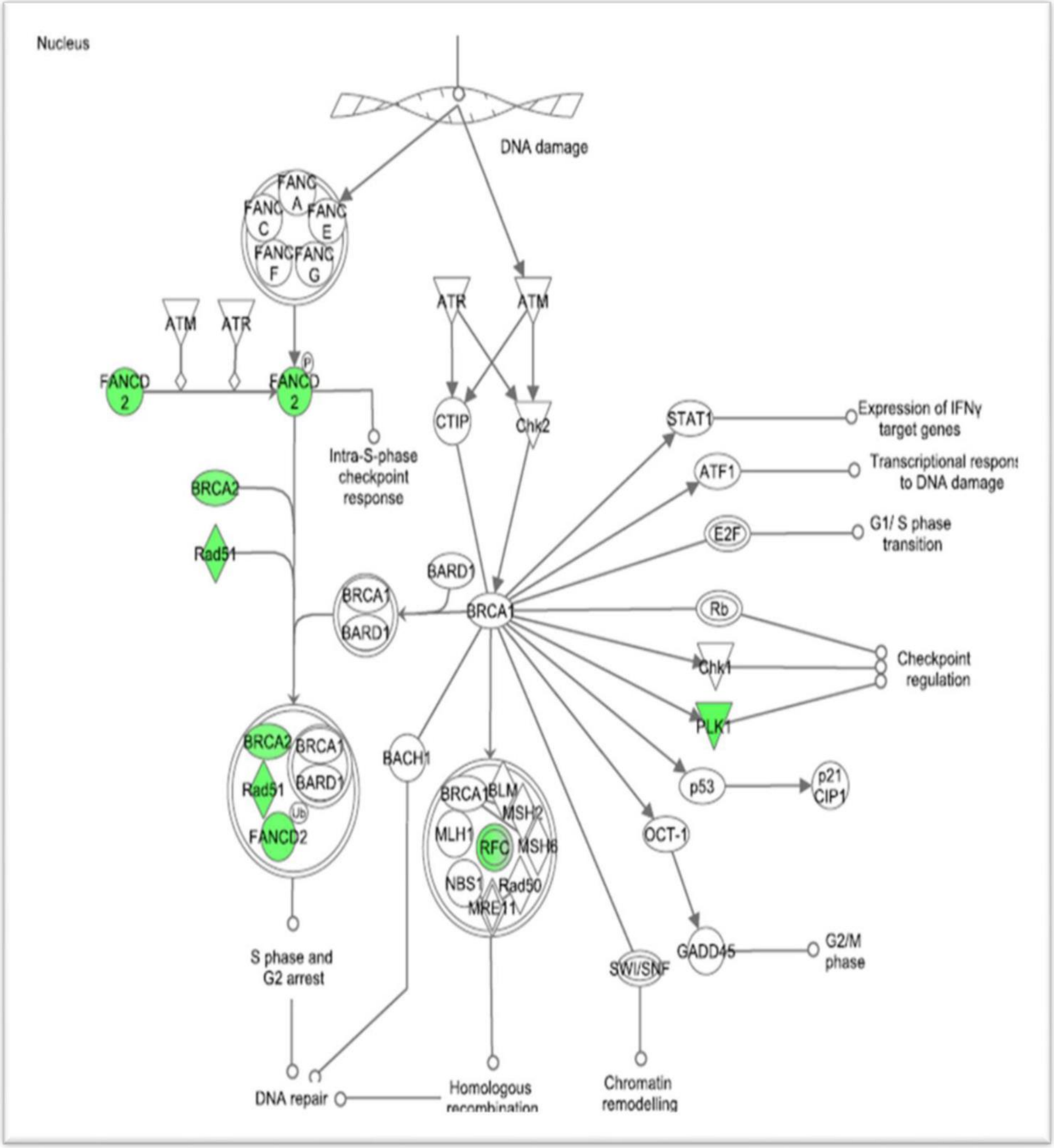

Supplementary Figure 3. Defective DNA repair and replication regulates senescence. IPA generated pathway shows decreased expression of the genes involved in DNA repair (RAD51, FANCD2, and BRCA2) and replication (RFC) during senescence. Green color denotes decreased expression of the genes.

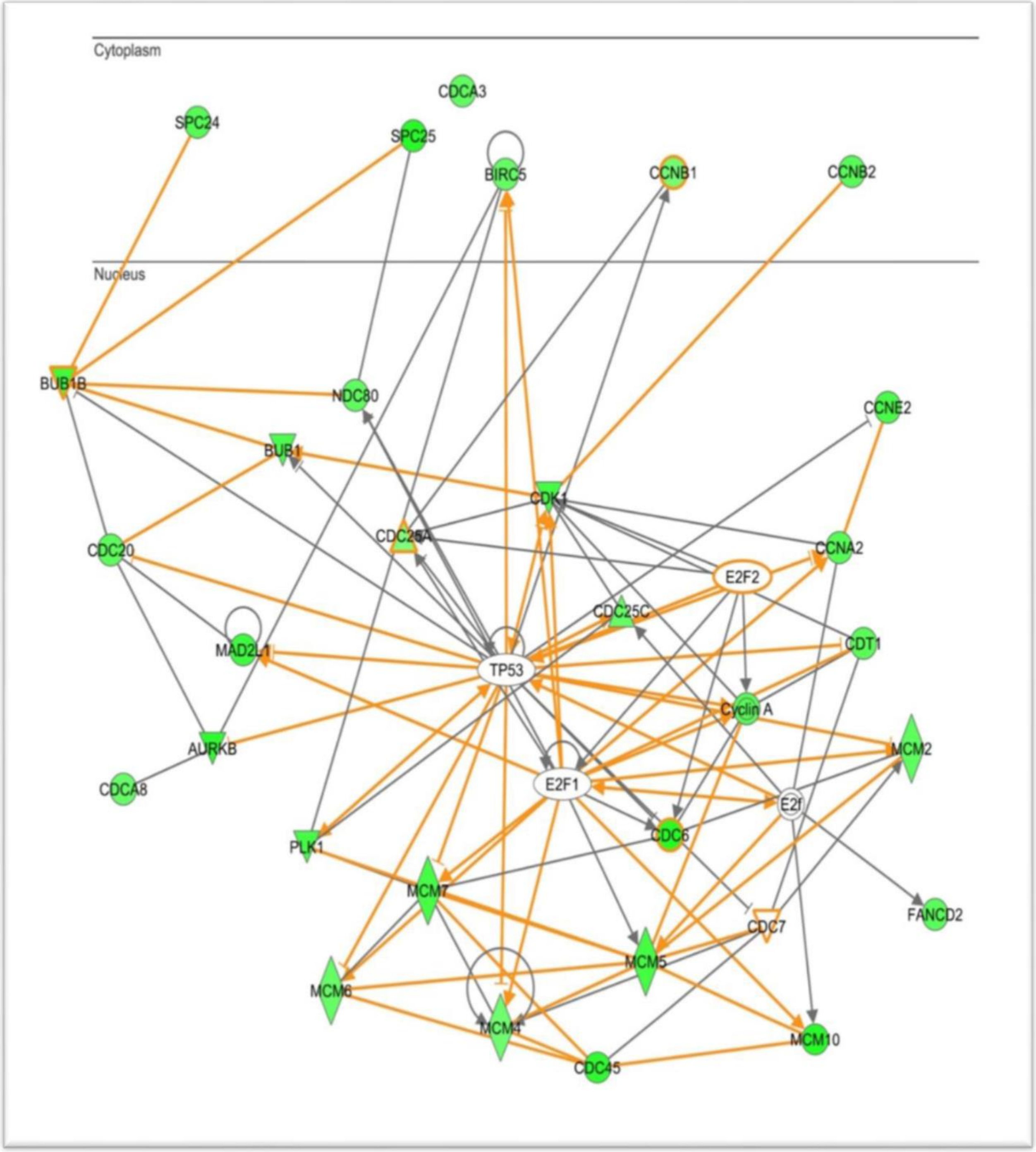

Supplementary Figure 4. Graphic representation of the network generated by IPA shows multiple downregulated genes during senescence in p53/RB-E2F pathway. Multiple genes downregulated during senescence are associated with the transcriptional network connected to p53-E2F. Nodes represent genes and lines show the relationship between genes. Green color represents decreased expression of the genes.

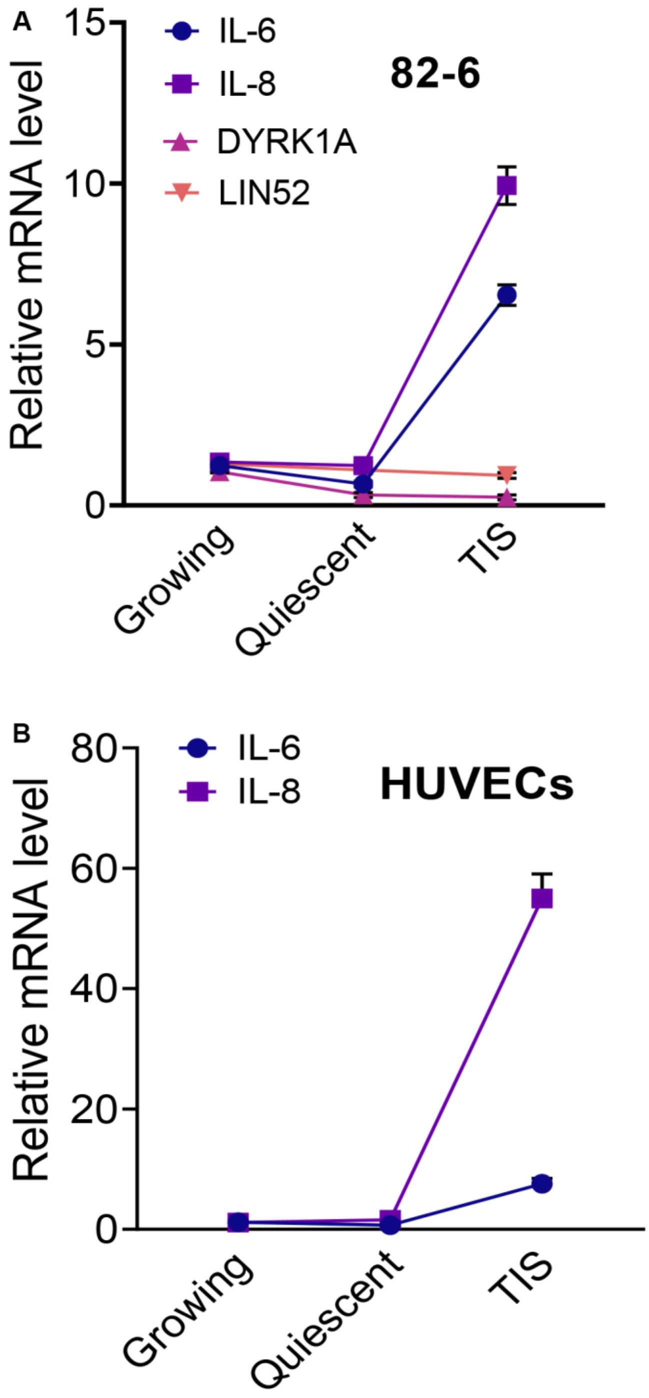

Supplementary Figure 5. Expression of SASP and DREAM complex associated genes in growing, quiescent and TNF-α senescent cells. (**A**) mRNA expression of IL-6, IL-8, DYRK1A, and LIN52 in growing, quiescent or TNF-α induced senescent 82-6 fibroblasts cells were measured using RT-PCR. (**B**) Expression of IL-6 and IL-8 in growing, quiescent, or TNF-α induced senescence HUVECs were measured using RT-PCR. GAPDH levels were used for normalization. Means ± SD are shown.
