# Supplementary material for: Key elements of cellular senescence involve transcriptional repression of mitotic and DNA repair genes through the p53-p16/RB-E2F-DREAM complex
Source: Aging (Albany NY). 2023 May 22;15(10):4012–34. doi: 10.18632/aging.204743 (PMC10258023; doi:10.18632/aging.204743)
Supplement: Supplementary Tables 2 and 3 [file aging-15-204743-s003.pdf]

Supplementary Table 2. List of antibodies used in this study.

Antibodies	Source	Catalog. no
BUBR1	BD	612503
MAD2L1	BD	610679
CDK1	Cell signaling	9112
CDK2	Cell signaling	2546
HEC1	Abcam	3613
SMC2	Abcam	Ab10399
FOXM1	Cell signaling	5436T
TNF-α	Cell signaling	11948
ACTIN	Sigma	A1978
TUBULIN	Sigma	T8328
Anti-mouse IgG, HRP-linked,Secondary Ab	Cell signaling	7076
Anti-rabbit IgG, HRP-linked, Secondary Ab	Cell signaling	7074

Supplementary Table 3. List of primers used for Q-PCR.

Primer	Gene	Catalog (Thermo fisher)
Hs00923894_m1	(P16) CDKN2A	4331182
Hs00355782_m1	(P21) CDKN1A	4331182
Hs00174103_m1	CXCL8 (IL-8)	4331182
Hs03929033_u1	IL-6	4331182
Hs00947993_m1	CDC25A	4448892
Hs01548894_m1	CDK2	4331182
Hs00153444_m1	PLK1	4331182
Hs00962413_m1	MCM6	4351372
Hs00608098_m1	E2F4	4331182
Hs00154374_m1	CDC6	4331182
Hs00153418_m1	RAD51	4331182
Hs00765700_m1	RBL1	4448892
Hs00176369_m1	DYRK1A	4331182
Hs00397517_m1	Lin 52	4331182
Hs01111968_m1	Lin 54	4448892
Hs01073585_m1	FOXM1	4448892
